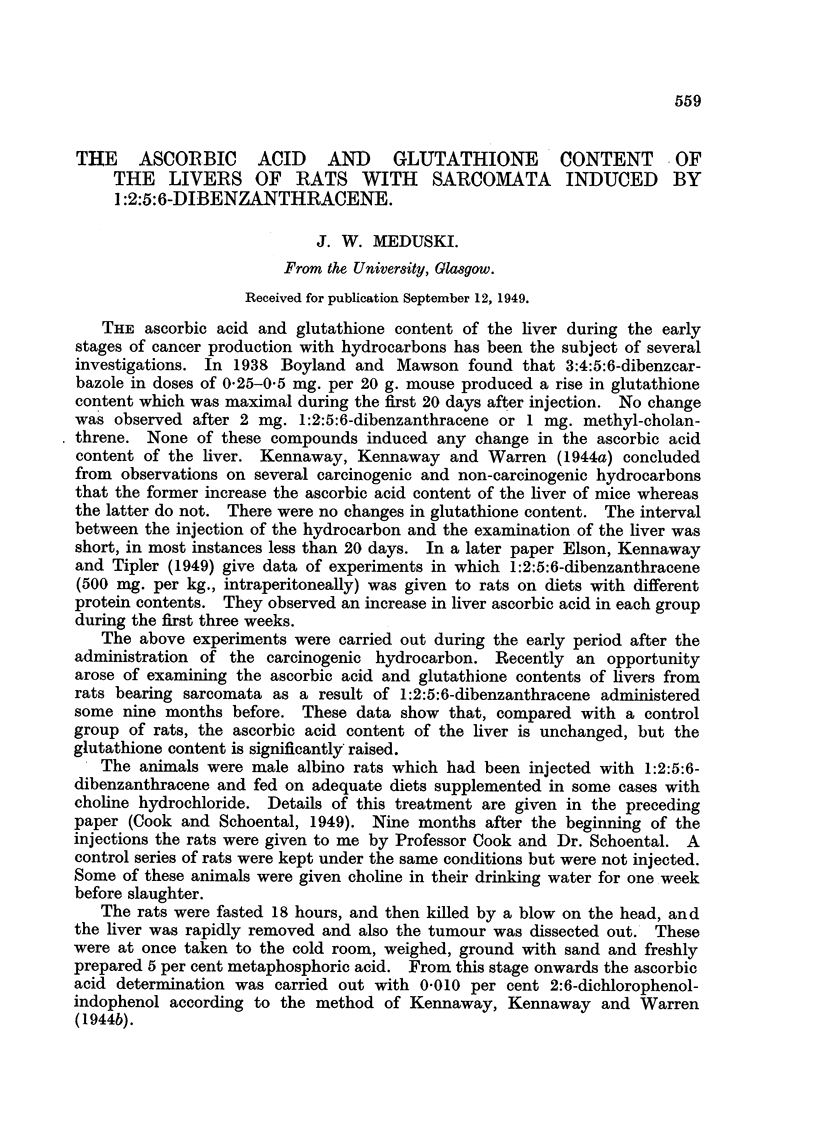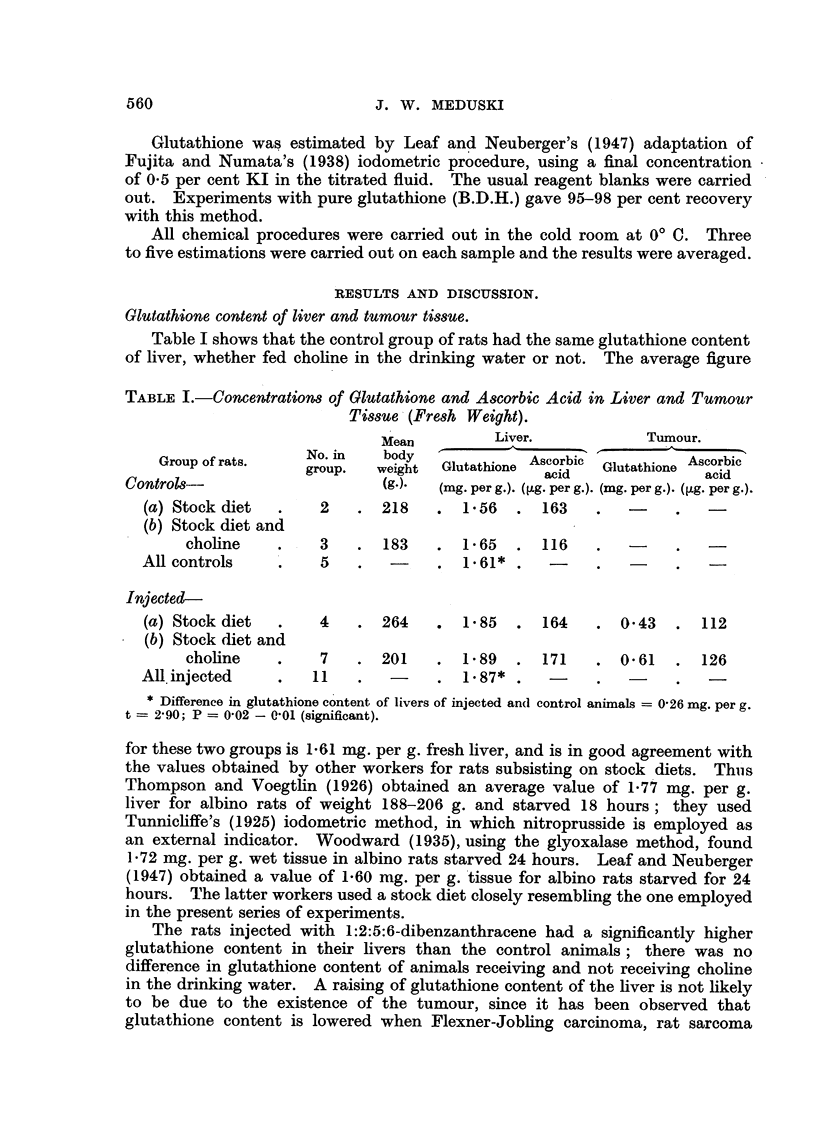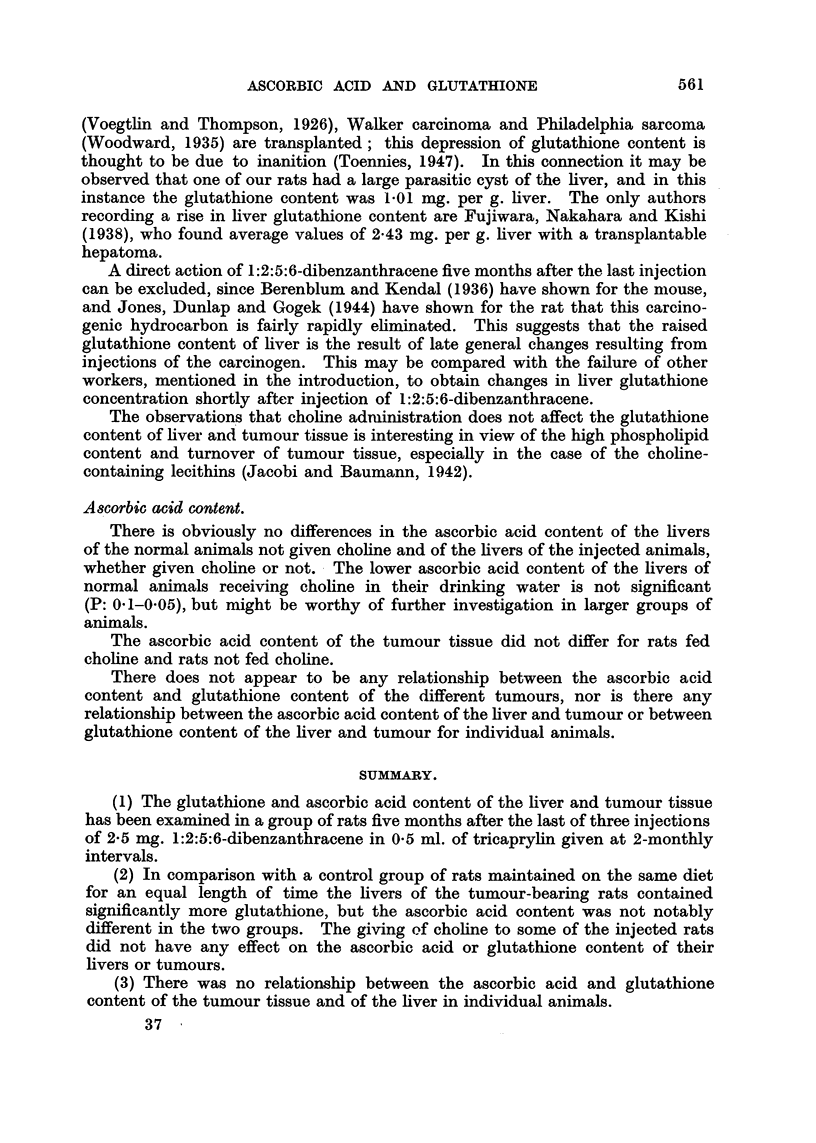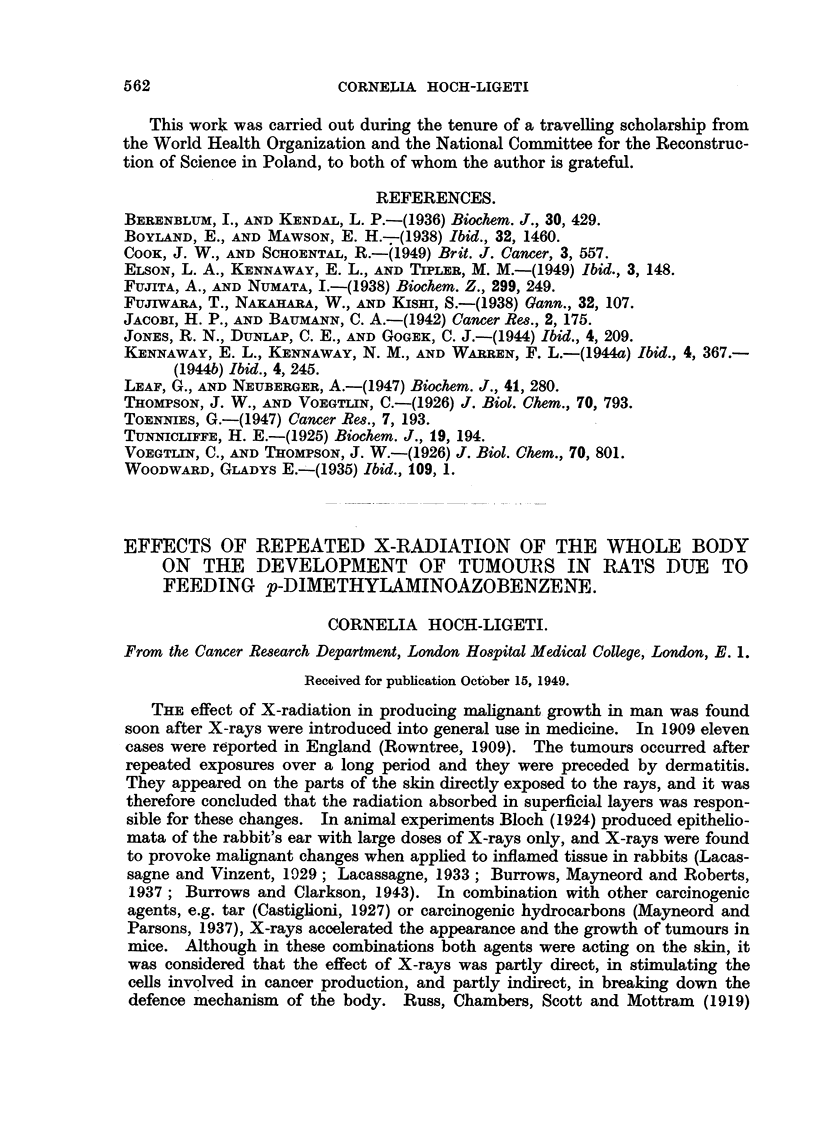# The Ascorbic Acid and Glutathione Content of the Livers of Rats with Sarcomata Induced by 1:2:5:6-Dibenzanthracene

**DOI:** 10.1038/bjc.1949.62

**Published:** 1949-12

**Authors:** J. W. Meduski


### THE ASCORBIC ACID AND GLUTATHIONE CONTENT OF THE LIVERS OF RATS WITH SARCOMATA INDUCED BY 1:2:5:6-DIBENZANTHRACENE.

J. W. MEDUSKI.

*From the University, Glasgow.*

Received for publication September 12, 1949.

THE ascorbic acid and glutathione content of the liver during the early stages of cancer production with hydrocarbons has been the subject of several investigations. In 1938 Boyland and Mawson found that 3:4:5:6-dibenzcarbazole in doses of 0·25–0·5 mg. per 20 g. mouse produced a rise in glutathione content which was maximal during the first 20 days after injection. No change was observed after 2 mg. 1:2:5:6-dibenzanthracene or 1 mg. methyl-cholanthrene. None of these compounds induced any change in the ascorbic acid content of the liver. Kennaway, Kennaway and Warren (1944*a*) concluded from observations on several carcinogenic and non-carcinogenic hydrocarbons that the former increase the ascorbic acid content of the liver of mice whereas the latter do not. There were no changes in glutathione content. The interval between the injection of the hydrocarbon and the examination of the liver was short, in most instances less than 20 days. In a later paper Elson, Kennaway and Tipler (1949) give data of experiments in which 1:2:5:6-dibenzanthracene (500 mg. per kg., intraperitoneally) was given to rats on diets with different protein contents. They observed an increase in liver ascorbic acid in each group during the first three weeks.

The above experiments were carried out during the early period after the administration of the carcinogenic hydrocarbon. Recently an opportunity arose of examining the ascorbic acid and glutathione contents of livers from rats bearing sarcomata as a result of 1:2:5:6-dibenzanthracene administered some nine months before. These data show that, compared with a control group of rats, the ascorbic acid content of the liver is unchanged, but the glutathione content is significantly raised.

The animals were male albino rats which had been injected with 1:2:5:6-dibenzanthracene and fed on adequate diets supplemented in some cases with choline hydrochloride. Details of this treatment are given in the preceding paper (Cook and Schoental, 1949). Nine months after the beginning of the injections the rats were given to me by Professor Cook and Dr. Schoental. A control series of rats were kept under the same conditions but were not injected. Some of these animals were given choline in their drinking water for one week before slaughter.

The rats were fasted 18 hours, and then killed by a blow on the head, and the liver was rapidly removed and also the tumour was dissected out. These were at once taken to the cold room, weighed, ground with sand and freshly prepared 5 per cent metaphosphoric acid. From this stage onwards the ascorbic acid determination was carried out with 0·010 per cent 2:6-dichlorophenol-indophenol according to the method of Kennaway, Kennaway and Warren (1944*b*).

Glutathione was estimated by Leaf and Neuberger's (1947) adaptation of Fujita and Numata's (1938) iodometric procedure, using a final concentration of 0·5 per cent KI in the titrated fluid. The usual reagent blanks were carried out. Experiments with pure glutathione (B.D.H.) gave 95–98 per cent recovery with this method.

All chemical procedures were carried out in the cold room at 0° C. Three to five estimations were carried out on each sample and the results were averaged.

### RESULTS AND DISCUSSION.

*Glutathione content of liver and tumour tissue.*

Table I shows that the control group of rats had the same glutathione content of liver, whether fed choline in the drinking water or not. The average figure

TABLE I.—*Concentrations of Glutathione and Ascorbic Acid in Liver and Tumour Tissue (Fresh Weight).*

| Group of rats. | No. in group. | Mean body weight (g.). | Liver. Glutathione (mg. per g.). | Liver. Ascorbic acid (μg. per g.). | Tumour. Glutathione (mg. per g.). | Tumour. Ascorbic acid (μg. per g.). |
|---|---|---|---|---|---|---|
| *Controls*— | | | | | | |
| (*a*) Stock diet | 2 | 218 | 1·56 | 163 | — | — |
| (*b*) Stock diet and choline | 3 | 183 | 1·65 | 116 | — | — |
| All controls | 5 | — | 1·61* | — | — | — |
| *Injected*— | | | | | | |
| (*a*) Stock diet | 4 | 264 | 1·85 | 164 | 0·43 | 112 |
| (*b*) Stock diet and choline | 7 | 201 | 1·89 | 171 | 0·61 | 126 |
| All injected | 11 | — | 1·87* | — | — | — |

* Difference in glutathione content of livers of injected and control animals = 0·26 mg. per g. $t = 2·90$; $P = 0·02 - 0·01$ (significant).

for these two groups is 1·61 mg. per g. fresh liver, and is in good agreement with the values obtained by other workers for rats subsisting on stock diets. Thus Thompson and Voegtlin (1926) obtained an average value of 1·77 mg. per g. liver for albino rats of weight 188–206 g. and starved 18 hours; they used Tunnicliffe's (1925) iodometric method, in which nitroprusside is employed as an external indicator. Woodward (1935), using the glyoxalase method, found 1·72 mg. per g. wet tissue in albino rats starved 24 hours. Leaf and Neuberger (1947) obtained a value of 1·60 mg. per g. tissue for albino rats starved for 24 hours. The latter workers used a stock diet closely resembling the one employed in the present series of experiments.

The rats injected with 1:2:5:6-dibenzanthracene had a significantly higher glutathione content in their livers than the control animals; there was no difference in glutathione content of animals receiving and not receiving choline in the drinking water. A raising of glutathione content of the liver is not likely to be due to the existence of the tumour, since it has been observed that glutathione content is lowered when Flexner-Jobling carcinoma, rat sarcoma

(Voegtlin and Thompson, 1926), Walker carcinoma and Philadelphia sarcoma (Woodward, 1935) are transplanted ; this depression of glutathione content is thought to be due to inanition (Toennies, 1947). In this connection it may be observed that one of our rats had a large parasitic cyst of the liver, and in this instance the glutathione content was 1·01 mg. per g. liver. The only authors recording a rise in liver glutathione content are Fujiwara, Nakahara and Kishi (1938), who found average values of 2·43 mg. per g. liver with a transplantable hepatoma.

A direct action of 1:2:5:6-dibenzanthracene five months after the last injection can be excluded, since Berenblum and Kendal (1936) have shown for the mouse, and Jones, Dunlap and Gogek (1944) have shown for the rat that this carcinogenic hydrocarbon is fairly rapidly eliminated. This suggests that the raised glutathione content of liver is the result of late general changes resulting from injections of the carcinogen. This may be compared with the failure of other workers, mentioned in the introduction, to obtain changes in liver glutathione concentration shortly after injection of 1:2:5:6-dibenzanthracene.

The observations that choline administration does not affect the glutathione content of liver and tumour tissue is interesting in view of the high phospholipid content and turnover of tumour tissue, especially in the case of the choline-containing lecithins (Jacobi and Baumann, 1942).

*Ascorbic acid content.*

There is obviously no differences in the ascorbic acid content of the livers of the normal animals not given choline and of the livers of the injected animals, whether given choline or not. The lower ascorbic acid content of the livers of normal animals receiving choline in their drinking water is not significant (P: 0·1–0·05), but might be worthy of further investigation in larger groups of animals.

The ascorbic acid content of the tumour tissue did not differ for rats fed choline and rats not fed choline.

There does not appear to be any relationship between the ascorbic acid content and glutathione content of the different tumours, nor is there any relationship between the ascorbic acid content of the liver and tumour or between glutathione content of the liver and tumour for individual animals.

### SUMMARY.

(1) The glutathione and ascorbic acid content of the liver and tumour tissue has been examined in a group of rats five months after the last of three injections of 2·5 mg. 1:2:5:6-dibenzanthracene in 0·5 ml. of tricaprylin given at 2-monthly intervals.

(2) In comparison with a control group of rats maintained on the same diet for an equal length of time the livers of the tumour-bearing rats contained significantly more glutathione, but the ascorbic acid content was not notably different in the two groups. The giving of choline to some of the injected rats did not have any effect on the ascorbic acid or glutathione content of their livers or tumours.

(3) There was no relationship between the ascorbic acid and glutathione content of the tumour tissue and of the liver in individual animals.

37

This work was carried out during the tenure of a travelling scholarship from the World Health Organization and the National Committee for the Reconstruction of Science in Poland, to both of whom the author is grateful.

### REFERENCES.

BERENBLUM, I., AND KENDAL, L. P.—(1936) *Biochem. J.*, **30**, 429.
BOYLAND, E., AND MAWSON, E. H.—(1938) *Ibid.*, **32**, 1460.
COOK, J. W., AND SCHOENTAL, R.—(1949) *Brit. J. Cancer*, **3**, 557.
ELSON, L. A., KENNAWAY, E. L., AND TIPLER, M. M.—(1949) *Ibid.*, **3**, 148.
FUJITA, A., AND NUMATA, I.—(1938) *Biochem. Z.*, **299**, 249.
FUJIWARA, T., NAKAHARA, W., AND KISHI, S.—(1938) *Gann.*, **32**, 107.
JACOBI, H. P., AND BAUMANN, C. A.—(1942) *Cancer Res.*, **2**, 175.
JONES, R. N., DUNLAP, C. E., AND GOGEK, C. J.—(1944) *Ibid.*, **4**, 209.
KENNAWAY, E. L., KENNAWAY, N. M., AND WARREN, F. L.—(1944*a*) *Ibid.*, **4**, 367.—(1944*b*) *Ibid.*, **4**, 245.
LEAF, G., AND NEUBERGER, A.—(1947) *Biochem. J.*, **41**, 280.
THOMPSON, J. W., AND VOEGTLIN, C.—(1926) *J. Biol. Chem.*, **70**, 793.
TOENNIES, G.—(1947) *Cancer Res.*, **7**, 193.
TUNNICLIFFE, H. E.—(1925) *Biochem. J.*, **19**, 194.
VOEGTLIN, C., AND THOMPSON, J. W.—(1926) *J. Biol. Chem.*, **70**, 801.
WOODWARD, GLADYS E.—(1935) *Ibid.*, **109**, 1.